# Financial perspective of private pharmacies in Tehran (Iran); is it a lucrative business?

**DOI:** 10.1186/2008-2231-20-62

**Published:** 2012-10-22

**Authors:** Khosro Keshavarz, Abbas Kebriaeezadeh, Amir Hashemi Meshkini, Shekoufeh Nikfar, Iman Mirian, Hasan Khoonsari

**Affiliations:** 1 Department of Pharmacoeconomics and Pharmaceutical administration, Faculty of pharmacy, Tehran University of Medical Sciences, Tehran, Iran; 2 Food & Drug Laboratory Research Center, Food & Drug Organization, Ministry of Health & Medical Education, Tehran, Iran; 3 Department of Public Health, Faculty of Public Health, Hormozqan University of Medical Sciences, Bandar abbas, Iran; 4 Department of Clinical Pharmacy, Faculty of Pharmacy, Tehran University of Medical Sciences, Tehran, Iran

**Keywords:** Pharmacy, Income, Cost and expense, Profitability, Dispensing fee, Iran

## Abstract

**Background and purpose of the study:**

Pharmacies as direct providers of medicine and pharmaceutical services to patients have an important role in the health status of a society. The assessment of their financial situations by healthcare policy makers is necessary to prevent any negative effects on population's health. In this study we aim to analyze the financial status of pharmacies in Tehran, Iran.

**Methods:**

This study is a cross-sectional study based on a survey. Two-hundred and eighty-eight private community daytime pharmacies in Tehran were selected by random sampling. We used two questionnaires to collect data regarding cost, expense and income factors of private pharmacies and the significance of each of them from these selected pharmacies. The data was collected in 2011 from Tehran pharmacies. Profitability of pharmacies in Tehran, Iran was calculated in its current situation and then estimated for three defined scenarios: 1. The dispensing fee is omitted (ceteris paribus), 2. Pharmacies are prohibited from selling hygienic & cosmetic products (ceteris paribus), 3. Scenarios 1 and 2 together (ceteris paribus). These data were analyzed by using SPSS and descriptive-analytic statistics.

**Results:**

About 68% of interviewees responded to our questionnaires. Our analysis indicated that the average annual costs (and expenses), income and profits of pharmacies are 73,181; 106,301; and 33,120 United States Dollar (USD), respectively. The analysis indicated that omission of dispensing fee (scenario 1) and prohibition of pharmacies from selling hygienic & cosmetic products (scenario 2) would decrease income of pharmacies to 18438 and 14034 USD/year, respectively. According to respondents, the cost (or expense) of properties and buildings, energy, taxes, delays in reimbursement by insurance companies, and renting the place of pharmacy could be considered as cost factors and prescription medicines, OTC medicines, dispensing fees, hygienic & cosmetic products, and long-term payment to pharmaceutical distribution companies as income factors, which have significant effects on a pharmacy's economy.

**Major conclusions:**

According to the results of this study, regarding the pharmacies' cost (and expenses) and incomes, the omission of dispensing fees for prescriptions has considerable negative effects on the profitability of pharmacies and likely on society's health.

## Introduction

Different countries use a variety of methods to organize their health systems. In these systems, the position of the pharmaceuticals is especially critical [1]. Pharmacies as direct providers of medicines and pharmaceutical services as well as potential centers for research in health services are one of the most important components of a health system [2]. Existence of private pharmacies in distribution network of medicines could provide good accessibility for medicines within the country [3,4]. Regarding to Iranian National Drug Policy (NDP) Private pharmacies belong to pharmacists who are allowed to establish their pharmacy under district regulation and legislation of Ministry of Health (MOH). They are responsible to provide medicines of National Drug List (NDL) for all patients and provide appropriated information according use of medicines [4]. Chain pharmacies and investment in multiple pharmacies are not allowed in Iran.

Nowadays, a wide range of services are provided by pharmacists, the results of which are an improvement in the overall health of a society. Studies have shown that the services provided by practical pharmacists create considerable improvement in health outcomes of society [5]. In addition, numerous studies show the role of pharmacists in diagnosing medical errors, medical interactions, and inappropriate prescribing [6-10]. Therefore, pharmacists have an important role in choosing the most appropriate, optimal and effective method of treatment [11].

In recent years, there has been controversy between policy makers and pharmacies regarding the omission of dispensing fees in Iran. Dispensing fee is a payment by patient to pharmacist after receiving prescription medicines to compensate pharmacy services including consultation, education on medicine usage and etc. The opposing sides have two different approaches. To solve the high amount of out-of-pocket payments by patients in Iran, some policymakers' approach is to omit the dispensing fee, given that it is not covered by most insurance companies. This approach is supported by some views in society regarding the extraordinary profit of pharmacies. In the second approach, pharmacists believe that the present situation of implemented policies in pricing areas, public pharmacy monopolies, internal inflation, etc. have caused many hardships, and the omission of the dispensing fee would lead to bankruptcy. Dispensing fee in Iran has two level: for prescriptions that their price are more than 0.8 USD it is about 0.75 USD and for prescription price under 0.8 USD it is 0.4 USD.

Currently in Iran, community pharmacies are divided into two types: public and private. According to the latest statistics from the Iranian Food and Drug Organization (2011), there are about 8,400 active pharmacies in Iran. Of these, 88% are privately owned and the rest are affiliated with public organizations or institutions. Although there are very few public pharmacies in Iran, they still have a large market share for many reasons, such as a monopoly in providing scarce and expensive medicines [12]. The pharmaceutical market has been extended by growth in GDP and health budgets but this extension had a small effect on health indicators [13]. Paying attention to the incentives for health service providers including pharmacies could improve health indicators.

Regarding the important role of pharmacies and their potential effects on the health of society, especially in low- and middle-income countries [14-17], all financial and non-financial incentives and disincentives regarding their role have to be monitored, and before any decisions are made the impacts of that policy on pharmacies' business has to be exactly analyzed.

In this study, we aim to analyze the financial situation and factors that affect the financial flow of private pharmacies and also attitudes of pharmacy owners regarding these factors to render a more clear understanding of the business aspects of pharmacies for policy makers.

## Method

This research is a cross-sectional study based on a survey, which was conducted in 2011 in two steps. In the first step, we tried to calculate the average profitability of private community pharmacies in Tehran based on data collected through questionnaires filled out by pharmacy owners. In this step, the profitability of a pharmacy was recalculated in three predefined scenarios to predict the situation of pharmacies after implementing such policies.

1. The dispensing fee is omitted (*ceteris paribus*)

2. Pharmacies are prohibited from selling hygienic & cosmetic products (*ceteris paribus*), [approximately all pharmacies in Iran provide hygienic & cosmetic products].

3. Scenarios 1 and 2 together (*ceteris paribus*)

In the second step, we aimed to achieve a general knowledge about the attitude of respondents toward the factors affecting the cost, expense and income components of pharmacies. The results of this step and the previous step were combined to suggest solutions for the current problems that face pharmacies and patients.

### Data collection measures

To evaluate the cost (and expense) and income information of pharmacies, the factors that affect the costs, expenses and income, and the importance of each factor from the pharmacy owners' point of view, two questionnaires were designed after a comprehensive literature review. The first was an open-ended and self-administered questionnaire in order to collect data from the pharmacy owners about costs, expenses and incomes of pharmacies. The second was a Likert-scaled questionnaire to collect information about the attitude of pharmacy managers toward the factors that affect the financial flow of a pharmacy. In both steps the data collection was designed to be through direct interviews. The validity and reliability of each questionnaire were checked by combination of expert opinion, pilot data collection from 10 pharmacies based on likert scale, and test-retest methods (r=0.83).

The respondents were free to refuse to answer these questionnaires, and they were assured before the interview that their identity would remain private.

### Sampling

Our population of interest included all of the private community pharmacies in Tehran (capital of Iran). According to Iranian Pharmacists' Association statistics (2011), there are 1,176 private pharmacies in Tehran, including 1,115 daytime pharmacies and 61 pharmacies open 24/7. As a sample, 228 pharmacies were selected by a random sampling method. Daytime pharmacies were only included in our study. For random selection we used random number table after assigning to all daytime pharmacies a number.

The respondents to the questionnaires were the pharmacy managers, whether they were pharmacists or not.

### Analysis

The analysis was conducted based on a typical daytime pharmacy, which was extracted from questionnaires. We used nonparametric techniques, including chi-square test by SPSS 18 software to analyze the data of study. To exchange IR Rial to US Dollar the official exchange rate declared by central bank (12260 IR Rials) was used.

The method of study was reviewed and approved by Institutional Review Board (IRB) of Tehran University of Medical Sciences (code of project: 900410216512).

## Results

About 68% of interviewees responded to our questionnaires. The results of our analysis is presented below.

### Part 1: Profitability of pharmacies

In Table 1, the general information regarding the number of pharmacies, margins and other needed data are collected. Knowing these data help Tables 2 and 3 to be better understood.

**Table 1:** **General information about Iran pharmaceutical market**^**12**^

**Total number of pharmacies**	**8393**^**a**^
profit margin of selling domestic drugs (2011)	20%
profit margin of selling imported drugs (2011)	10-15%
The average profit gained from distribution companies discount	3%
percentage of expired drugs in pharmacies	1%

**Table 2:** The cost and expense components of pharmacies in 2011

**Cost and Expense items**		**Amount (USD)**	**Min & Max (USD)**	**Details (according to questionnaire information and experts attitude**
Fixed	The average of depreciation expense of properties^b^	1697	(821 and 2049)	The average, Min and Max properties price is obtained from questionnaire information and market price
Variable	The average manpower expense	38203	(31593 and 44814)	The average is based on four personnel: pharmacist, pharmacy technician, cashier, hygienic salesman. Max: 5 and Min: 3 personnel are included
	The average expense of renting pharmacy location	19576	(9788 and 48940)	The average of monthly renting expense is 1631 USD (Max: 4078 USD and Min: 816 USD)
	The average expense of consumed energy (electricity, gas, water, telephone)	1958	(1468 and 2936)	The average of monthly consumed energy expense is 163 USD (Max: 245 USD and Min: 122 USD)
	The average expense of consumables	1468	(979 and 1958)	The averageexpense of monthly consumablesis 122 USD/year (Max: 163 and Min: 82 USD/year)
	Annual tax expense	2447	(1631 and 3263)	The average expense of yearly tax is 2447 USD/year (Max: 3263 USD/year and Min: 1631 USD/year)
	The average of imposed expenses and costs during activities (insurance deductions, drug expiration, drug waste)	1958	(979 and 2936)	The average expense of monthly imposed is 163 USD/year (Max: 245 USD/year and Min: 82 USD/year)
	The average expenses of delays in insurance companies reimbursements	3467	(2600 and 4333)	It is calculated based on %17 bank profits. The average delay is considered as 4 months (Max: 5 months and Min: 3 months)
	The average expense of entering prescription data on computer	1918	(343 and 3837 )	To register each prescription .07 USD/year and 24.5 days in a month is calculated. The average 100 prescriptions in a day are included (Max 200 and Min 70 prescriptions
	The average of other expenses (advertisement, repair and maintenance, internet, personnel food, software updates, transportation)	489	(294 and 979)	the average is 408 USD/year (Max: 979 USD/year and Min 82 USD/year)
	The average total expense of a pharmacy (2011)	**73181**		

**Table 3:** The income components of pharmacies in 2011

**Income items**	**Amount**	**Max & Min**	**Details (according to questionnaire information and experts attitude)**
	**(USD/year)**	**(USD/year)**	
The average net income gained from selling prescription medicines	27732	(11093 and 55465)	The net income is calculated based on averagely 17% margin
The average net income gained from selling OTC drugs	18597	(14682 and 36705)	The net income is calculated based on averagely 19% margin
The average net income gained from selling hygienic & cosmetic products	19086	(14682 and 36705)	The net income is calculated based on averagely 18% averagely margin
The average net income gained from dispensing fees	14682	(12235 and 23246)	It is calculated based on averagely 49 USD/year
The average net income gained from discounts and distribution companies prizes	13051	(979 and 19576)	It is calculated based on 5% discounts
The average net income gained from purchases with long-term payments	5200	(1733 and 8666)	It is calculated based on average reimbursement of 3 months (Max: 5months and Min: 1 month). Also bank profit in 2011 supposed to be 17%
The average net income gained from increase of drug prices	7953	(3670 and 12235)	It is calculated based on the average stock of 122349 USD/year and 3%-10% increase in prices
The average total income of a pharmacy (2011)	**106301**		

In Table 2, all the detailed information about the cost (and expense) components of pharmacies are presented. It shows that an average cost and expense of a daytime pharmacy is 73,181 USD/year. Of this, 52% of expenses relate to manpower, 26.8% relate to renting the pharmacy building, and the remainder (21.2%) relate to other expenses.

In Table 3, all income components of a typical pharmacy are presented. The table shows that an average annual income of a pharmacy equals 106,301 USD. This income relates to prescription medicines (26.1%), over-the-counter (OTC) drugs (17.5%), hygienic & cosmetic products and orthopedics (18%), and dispensing fees (13.8%). The results of Tables 2 and 3 show that the average profit of a pharmacy equals 33,120 USD annually and 2,760 USD monthly. This monthly amount varies from 1631.3 (Min) to 4894 (Max).

#### Scenario 1. The dispensing fee is omitted (ceteris paribus)

The results of the tables show that if the dispensing fee is omitted, the average pharmacy income would be decreased to 91,619 USD/year. If we deduct the costs and expenses of pharmacy from this amount, the net income of a pharmacy equals 18,438 USD in a year and 1,536.5 USD in a month (44% reduction).

#### Scenario 2. Pharmacies are prohibited from selling hygienic & cosmetic products (ceteris paribus)

If the dispensing fee exists but the sale of hygienic & cosmetic products is omitted, the pharmacy income would be decreased to 87,215 USD/year. If we deduct costs and expenses of pharmacy from this amount, the net income of a pharmacy equals 14,034 USD per year and 1,169.5 USD per month (58% reduction).

#### Scenario 3. Scenarios 1 and 2 together (ceteris paribus)

If both the dispensing fees and hygienic & cosmetic products are omitted and only the income of medicine sales is calculated, then the pharmacy would generate an income of 72533 USD, resulting in a net income of 648 USD and 54 USD lost annually and monthly, respectively (98% reduction).

### Part 2: The factors affecting the cost, expense and income components of pharmacy business

In this part, the results of responders' attitude toward the significance of effective factors on a pharmacy's economic improvement are shown.

1. Factors affecting pharmacy costs: To answer this question, two fixed factors and nine variable factors were defined. In Table 4, the results of the statistical analysis are presented to answer this question.

**Table 4:** the results of statistical analysis (the attitude of pharmacists about the factors relating the expenses of pharmacy)

**Variables**		**Responders attitude**					**Degree of freedom (df)**	**chi square (*X***^***2***^**)**	**Significance**
		**Strongly agree**	**agree**	**neutral**	**disagree**	**Strongly disagree**			
Fixed	properties	63	164	38	12	11	4	277.521	0
	building	113	130	23	14	8	4	240.785	0
Variable	manpower	156	85	24	14	9	4	274.743	0
	consumables	45	66	62	55	60	4	4.535	0.338
	consumed energy	61	78	61	41	47	4	14.361	0.006
	renting location	65	74	62	41	46	4	13.076	0.011
	tax	38	93	60	49	48	4	31.41	0
	insurance deductions	50	71	62	50	55	4	5.576	0.233
	drug expiration	47	75	62	49	55	4	8.944	0.063
	delay in reimbursement	70	83	58	42	35	4	26.965	0
	description registration	44	60	72	60	52	4	7.556	0.109

As Table 4 shows, according to respondents' views, the most significant factors affecting a pharmacy's costs and expenses are the properties and building (as fixed costs); the manpower, consumed energy, tax, and delays in reimbursement by insurance companies to pharmacies (most of insurance organization reimburse pharmacies after several months from providing medicines to patient); and renting the pharmacy location. However, the consumed materials, insurance deductions, drug expiration, and entering prescription data into a computer program (some pharmacies doesn't have in-time prescription data entering in system and have to pay for that) do not have a significant effect on pharmacy costs and expenses.

2. Factors affecting pharmacies' income: To answer this question, seven factors are defined. In Table 5, statistical analysis results are presented to answer this question.

**Table 5:** the results of statistical analysis (attitude of pharmacists about the factors relating the income of pharmacies)

**Variables**	**Responders attitude**					**Degree of freedom (df)**	**chi square (*X***^***2***^**)**	**Significance (sig)**
	**Strongly agree**	**agree**	**neutral**	**disagree**	**Strongly disagree**			
Description	173	70	22	13	10	4	329.743	0
OTC	82	162	28	9	7	4	33.229	0
dispensing fee	171	85	19	7	6	4	352.833	0
cosmetic hygienic products	103	142	21	14	8	4	258.424	0
company discounts	40	56	72	62	58	4	9.361	0.053
increase in drug prices	44	65	67	53	59	4	6.097	0.192
A	78	162	32	9	7	4	293.285	0

As the results in Table 5 show, according to responders' views, the income earned from the sale of prescription medicines, OTC medicines, dispensing fees, hygienic & cosmetic products and long-term payment of purchasing from distribution companies have a significant effect on pharmacies' income (p < 0.01), but other factors, such as the income gained from pharmaceutical company discounts and increases in drug prices, do not have significant effects on pharmacies' income (p > 0.05).

3. Solutions to improve pharmacies' economy: To answer this question, nine factors were defined. In Table 6, the statistical analysis results are presented to answer this question.

**Table 6:** the results of statistical analysis (the ways to improve business of pharmacy)

**Variables**	**Responders attitude**					**Degree of freedom (df)**	**chi square (*X***^***2***^**)**	**Significance (sig)**
	**Strongly agree**	**agree**	**neutral**	**disagree**	**Strongly disagree**			
tax exemption	82	169	21	9	7	4	334.5	0
exclusive selling of some cosmetic-hygienic products	64	169	30	13	12	4	33.021	0
elimination of monopolies regarding providing medicines	93	156	21	11	7	4	295.264	0
drug margin increase	88	161	23	9	7	4	307.903	0
coverage of drug items	64	162	37	13	12	4	267.938	0
increasing the share of insurance companies	40	56	72	62	58	4	9.361	.053
quick insurance reimbursements	80	164	27	12	5	4	305.646	0
dispensing fee	68	165	32	13	10	4	287.382	0
loans	93	149	28	10	8	4	264.049	0

As Table 6 shows, according to responders' views, tax exemption, selling of hygienic & cosmetic products, scarce and exclusive medicine (in Iran some medicines like opioids, and very expensive anti cancers and etc. are only available in public pharmacies), medicine margin, insurance coverage of medicines, quick insurance reimbursement, dispensing fees, and financial support (p < 0.01) are the most effective methods of pharmacies' financial improvement. However, the increase in share of insurance from drug costs is not effective on pharmacies' financial improvement (p > 0.05), from the pharmacy owners' points of view.

## Discussion

According to the results, our study showed that the average profit of a typical pharmacy in Tehran is 2,760 USD per month. Also, our analysis in the second part showed that some factors—including tax exemptions, sale of hygienic & cosmetic products, coverage of medicines by insurance companies, quick insurance reimbursement, dispensing fees, and appropriate financial incentives—have a greater effect on offering pharmaceutical services and also pharmacy income.

This is the first comprehensive study about business-related issues of pharmacies in Iran, which encompasses most of the expense (and cost) and income components of pharmacies as well as the effective factors on the profitability of pharmacies from pharmacists' and pharmacy owners' points of view. Similar studies from other countries were also not found.

Our research seems to be consistent with Pourheidari's study (2010) in which the pharmacy income was only slightly more than its costs and expenses [18]. This result is also in conflict with some policymakers' views about the extraordinary profitability of pharmacies. It also should be mentioned that to establish a pharmacy and enter the market, a large amount of investment is needed in advance (an average investment to establish a pharmacy in Tehran is estimated to be 264698 USD). In this study, however, only the active pharmacies were taken into account in order to calculate their fixed and variable costs and expenses, so we neglected establishment costs. On the other hand, regarding the social responsibility and important role of pharmacies, the difference between cost and income does not seem to be appropriate because this calculated profit encompasses some nonprofessional service provision, including sale of hygienic & cosmetic products. If pharmacies were prohibited from providing such services, their profitability would likely decrease, as the scenarios showed. From this aspect, our study is similar to a study by Mohammadzade (2010), indicated that pharmacies are not profitable but even disadvantageous if they only provide medicines [19].

The evidence showed that pharmacy margins have an important role in whether or not pharmacists convince patients to use brand or generic medicines [20]. Therefore, it must be mentioned by policymakers that decreasing pharmacies' profitability by cost containment or any other strategy may lead the pharmacies to compensate their profit through other ways, either legal or illegal, including selling non-OTC medicines without prescription, imposing unnecessary costs to patients, etc., that can offset those policies and sometimes threaten the society's health. A study regarding European Union (EU) countries' pharmaceutical policies after the economic crisis indicated that pharmacies, as one of the important stakeholders in the healthcare system, were taken into consideration in designing such cost-controlling policies [21]. We must note that our results were concluded regardless of the appropriateness or weaknesses of the current implemented policies, including dispensing fees, and they must be evaluated in term of equity, efficiency, patient satisfaction, and other issues in further studies.

Another point is about the effect of insurance coverage level on pharmacies' business. However, the coverage of medicines by insurance, probably because of its effect of increasing demand, was considered as an effective factor on profitability of pharmacies from the pharmacy owners' point of view, but the increase in share of insurance from pharmaceutical costs was not identified by them as a significantly effective factor. This could be justified by reasons including the irrational delays by insurance in reimbursement to pharmacies. The studies also showed that the profitability of prescription medicines has decreased [22]; a primary reason could be the increasing contribution of insurance on prescription price [11,22].

### Limitations

In this study we were faced with limited access to data, given that most pharmacists and pharmacy owners were reluctant to disclose their business-related data. The expense (and cost) and income data were collected in a self-reported way, and the validity of their claims could not be evaluated.

## Conclusion

It can be concluded that there is a gap in the current financial situation of pharmacies in Tehran of optimal status in terms of profitability. This situation would be worse, however, if the considered scenarios (omission of dispensing fees and sales of hygienic & cosmetic products) are implemented.

## Endnotes

^a^ According to Food and Drug Organization about 95% of them have private ownership and only 5% are public

^b^ The Straight-line depreciation method was used to compute depreciation of assets in pharmacies

## Competing interests

One of the authors, H. Khoonsari is a pharmacist and pharmacy owner.

## Authors' contributions

AK: designing the research method, final approval of results. KK: data collection, analyzing collected data, contribution in designing research method. AHM: data collection, analyzing collected data, revising the manuscript. SN: designing the research method. IM: analyzing the collected data, drafting the manuscript. HK: data collection, contribution in designing research method. All authors read and approved the final manuscript.
